# [μ-10,22-Dichloro-3,6-bis­(2-furylmeth­yl)-3,6,14,18-tetra­azatricyclo­[18.3.1.1^8,12^]penta­cosa-1(23),8,10,12(25),13,18,20(24),21-octa­ene-24,25-diolato-κ^8^ *N* ^3^,*N* ^6^,*O* ^24^,*O* ^25^:*N* ^14^,*N* ^18^,*O* ^24^:*O* ^25^]bis­[chloridocopper(II)] acetonitrile solvate

**DOI:** 10.1107/S1600536809000166

**Published:** 2009-01-08

**Authors:** Chen Chen, Yu Cheng, Pan Liu, Hong Zhou, Zhi-Quan Pan

**Affiliations:** a Key Laboratory for Green Chemical Process of Ministry of Education, Wuhan Institute of Technology, Wuhan 430073, People’s Republic of China

## Abstract

The title compound, [Cu_2_(C_31_H_30_Cl_2_N_4_O_4_)Cl_2_]·CH_3_CN, was synthesized by cyclo­condensation between *N*,*N*′-bis­(2-fur­yl)-*N*,*N*′-bis­(3-formyl-5-chloro­salicylaldehyde)ethyl­enediamine and 1,3-diamino­propane in the presence of Cu^II^ ions. It is an unsymmetrical dinuclear Cu^II^ complex. The coordination geometry for each Cu^II^ atom can be discribed as distorted square-pyramidal. The two Cu atoms are bridged by two phenolate O atoms with a Cu⋯Cu distance of 3.0274 (9) Å.

## Related literature

For general background, see: Hori *et al.* (2001 ▶); Karunakaran & Kandaswamy (1994 ▶); McCollum *et al.* (1994 ▶); Okawa *et al.* (1998 ▶); Sun *et al.* (2001 ▶). For the synthesis of *N*,*N*′-bis­(2-fur­yl)-1,2-diamino­ethane, see: Rameau (1938 ▶).
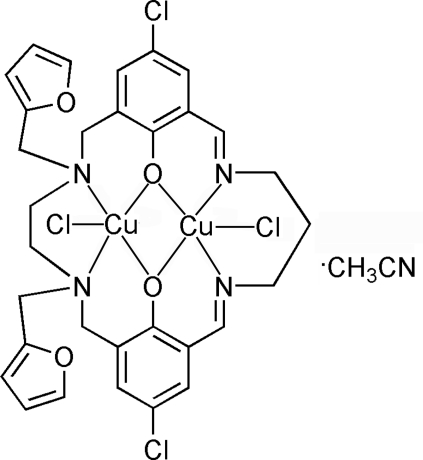


## Experimental

### 

#### Crystal data

[Cu_2_(C_31_H_30_Cl_2_N_4_O_4_)Cl_2_]·C_2_H_3_N
*M* *_r_* = 832.52
Triclinic, 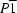

*a* = 10.4439 (19) Å
*b* = 13.083 (4) Å
*c* = 14.319 (3) Å
α = 112.039 (3)°
β = 100.290 (4)°
γ = 98.259 (3)°
*V* = 1736.2 (7) Å^3^
*Z* = 2
Mo *K*α radiation
μ = 1.58 mm^−1^
*T* = 291 (2) K
0.30 × 0.26 × 0.24 mm

#### Data collection

Bruker APEX CCD diffractometer
Absorption correction: multi-scan (*SADABS*; Bruker, 2001 ▶) *T*_min_ = 0.63, *T*_max_ = 0.69
9923 measured reflections
6662 independent reflections
4514 reflections with *I* > 2σ(*I*)
*R*_int_ = 0.036

#### Refinement

*R*[*F* ^2^ > 2σ(*F* ^2^)] = 0.054
*wR*(*F* ^2^) = 0.111
*S* = 1.01
6662 reflections
434 parameters
H-atom parameters constrained
Δρ_max_ = 0.51 e Å^−3^
Δρ_min_ = −0.45 e Å^−3^

### 

Data collection: *SMART* (Bruker, 2007 ▶); cell refinement: *SAINT* (Bruker, 2007 ▶); data reduction: *SAINT*; program(s) used to solve structure: *SHELXTL* (Sheldrick, 2008 ▶); program(s) used to refine structure: *SHELXTL*; molecular graphics: *SHELXTL*; software used to prepare material for publication: *SHELXTL*.

## Supplementary Material

Crystal structure: contains datablocks global, I. DOI: 10.1107/S1600536809000166/hy2178sup1.cif

Structure factors: contains datablocks I. DOI: 10.1107/S1600536809000166/hy2178Isup2.hkl

Additional supplementary materials:  crystallographic information; 3D view; checkCIF report

## Figures and Tables

**Table 1:** Selected bond lengths (Å)

Cu1—Cl1	2.5022 (13)
Cu1—O1	1.974 (3)
Cu1—O2	1.987 (3)
Cu1—N3	1.971 (4)
Cu1—N4	1.984 (4)
Cu2—Cl2	2.3104 (13)
Cu2—O1	1.940 (3)
Cu2—O2	2.010 (2)
Cu2—N1	2.104 (3)
Cu2—N2	2.047 (3)

**Fig. 1.**
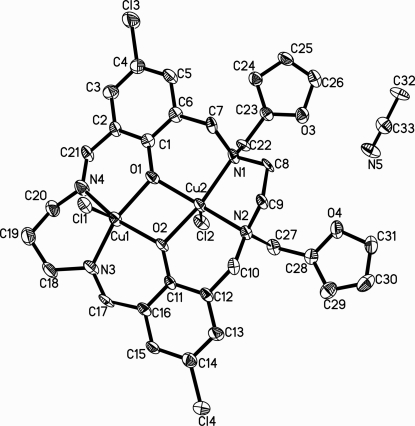
Molecular structure of the title compound. Displacement ellipsoids are drawn at the 30% probability level. H atoms have been omitted for clarity.

## supplementary crystallographic information

### Comment

The design and synthesis of phenol-based macrocyclic ligands with N(amino)_2_O_2_ and N(imino)_2_O_2_ metal-binding sites sharing two phenolate O atoms have drawn increasing attention for their potential unique properties (Hori *et al.*, 2001; Karunakaran & Kandaswamy, 1994;  McCollum *et al.*, 1994; Okawa *et al.*, 1998; Sun *et al.*, 2001). In this paper, we report a new unsymmetrical homodinuclear complex of N(amino)_2_N(imino)_2_O_2_-type macrocycle.

The structure of the title compound is shown in Fig. 1. Tne Cu1 atom is five-coordinated by two imino N atoms and two phenolate O atoms from the macrocyclic ligand and one Cl atom. The Cu2 atom is also five-coordinated by two amino N atoms and two phenolate O atoms from the macrocyclic ligand and one Cl atom. The coordination geometry for each Cu^II^ atom can be described as distorted square-pyramidal. The basal plane of Cu1 is composed of N3, N4, O1, O2 with a mean plane deviation of 0.0096 Å. The distances between Cu1 and the coordinated atoms in the basal plane are in a range of 1.971 (4)–1.987 (3) Å (Table 1). The mean plane deviation of the basal plane of Cu2 composed of N1, N2, O1, O2 is 0.0185 Å, with the distances between Cu2 and coordinated atoms in the basal plane in a range of 1.940 (3)–2.104 (3) Å. The difference in the distances of Cu1–coordinated atoms and Cu2–coordinated atoms is attributed to the dissimilar size of imino and amino groups. The two Cu atoms are bridged by two phenolate O atoms from the macrocyclic ligand. Two Cl atoms occupy the axial positions, respectively.

### Experimental

N,N'-bis(2-furyl)-1,2-diaminoethane was prepared using a variant of the method suggested by Rameau (1938). The precursor ligand N,N'-bis(2-furyl)-N,N'-bis(3-formyl-5-chlorosalicylaldehyde)ethylenediamine (H_2_*L*) was prepared through the Mannich reaction between 5-chlorosalicyladehyde (0.2 mol), polyformaldehyde (0.2 mol) and N,N'-bis(2-furyl)-1,2-diaminoethane (0.1 mol). The title compound was synthesized by stepwise template method through the reaction of the methanol solution of H_2_*L* (0.5 mmol) with the methanol solution of 1,3-diaminopropane (0.5 mmol), Cu(CH_3_CO_2_)_2_.H_2_O (0.5 mmol), and NiCl_2_.6H_2_O (0.5 mmol). The blue crystals of the title compound suitable for X-ray diffraction were obtained by the evaporation of the mother solution in about a month.

### Refinement

H atoms were positioned geometrically and refined as riding atoms, with C—H = 0.93 (CH), 0.97 (CH_2_) and 0.96 (CH_3_) Å and with *U*_iso_(H) = 1.2(or 1.5 for methyl)*U*_eq_(C).

### Crystal data

**Table:**

[Cu_2_(C_31_H_30_Cl_2_N_4_O_4_)Cl_2_]·C_2_H_3_N	*Z* = 2
*M**_r_* = 832.52	*F*(000) = 848
Triclinic, *P*1	*D*_x_ = 1.593 Mg m^−^^3^
Hall symbol: -P 1	Mo *K*α radiation, λ = 0.71073 Å
*a* = 10.4439 (19) Å	Cell parameters from 2324 reflections
*b* = 13.083 (4) Å	θ = 2.2–25.3°
*c* = 14.319 (3) Å	µ = 1.58 mm^−^^1^
α = 112.039 (3)°	*T* = 291 K
β = 100.290 (4)°	Block, blue
γ = 98.259 (3)°	0.30 × 0.26 × 0.24 mm
*V* = 1736.2 (7) Å^3^	

### Data collection

**Table:**

Bruker APEX CCD diffractometer	6662 independent reflections
Radiation source: sealed tube	4514 reflections with *I* > 2σ(*I*)
graphite	*R*_int_ = 0.036
φ and ω scans	θ_max_ = 26.0°, θ_min_ = 2.0°
Absorption correction: multi-scan (*SADABS*; Bruker, 2001)	*h* = −12→12
*T*_min_ = 0.63, *T*_max_ = 0.69	*k* = −13→16
9923 measured reflections	*l* = −17→15

### Refinement

**Table:**

Refinement on *F*^2^	Primary atom site location: structure-invariant direct methods
Least-squares matrix: full	Secondary atom site location: difference Fourier map
*R*[*F*^2^ > 2σ(*F*^2^)] = 0.054	Hydrogen site location: inferred from neighbouring sites
*wR*(*F*^2^) = 0.111	H-atom parameters constrained
*S* = 1.01	*w* = 1/[σ^2^(*F*_o_^2^) + (0.05*P*)^2^ + 0.55*P*] where *P* = (*F*_o_^2^ + 2*F*_c_^2^)/3
6662 reflections	(Δ/σ)_max_ < 0.001
434 parameters	Δρ_max_ = 0.51 e Å^−^^3^
0 restraints	Δρ_min_ = −0.45 e Å^−^^3^

### Fractional atomic coordinates and isotropic or equivalent isotropic displacement parameters (Å^2^)

**Table:**

	*x*	*y*	*z*	*U*_iso_*/*U*_eq_	
C1	−0.0844 (4)	0.8642 (3)	0.2679 (3)	0.0342 (9)	
C2	−0.1625 (5)	0.9063 (3)	0.3379 (3)	0.0383 (9)	
C3	−0.2650 (5)	0.8331 (4)	0.3466 (4)	0.0542 (12)	
H3	−0.3152	0.8609	0.3940	0.065*	
C4	−0.2920 (5)	0.7183 (4)	0.2841 (4)	0.0524 (12)	
C5	−0.2168 (5)	0.6747 (4)	0.2146 (4)	0.0495 (11)	
H5	−0.2359	0.5972	0.1737	0.059*	
C6	−0.1104 (5)	0.7485 (4)	0.2057 (3)	0.0398 (10)	
C7	−0.0367 (4)	0.6997 (3)	0.1234 (3)	0.0357 (9)	
H7A	−0.0698	0.6181	0.0896	0.043*	
H7B	−0.0558	0.7293	0.0709	0.043*	
C8	0.1739 (5)	0.6779 (3)	0.0826 (3)	0.0379 (9)	
H8A	0.1092	0.6142	0.0261	0.045*	
H8B	0.2481	0.6497	0.1069	0.045*	
C9	0.2251 (4)	0.7658 (3)	0.0416 (3)	0.0335 (9)	
H9A	0.2765	0.7339	−0.0078	0.040*	
H9B	0.1495	0.7838	0.0057	0.040*	
C10	0.3374 (4)	0.9594 (3)	0.0903 (3)	0.0325 (9)	
H10A	0.2566	0.9561	0.0427	0.039*	
H10B	0.4039	0.9427	0.0508	0.039*	
C11	0.3021 (4)	1.1280 (3)	0.2317 (3)	0.0338 (8)	
C12	0.3870 (4)	1.0792 (3)	0.1723 (3)	0.0362 (9)	
C13	0.5049 (5)	1.1461 (4)	0.1761 (4)	0.0490 (12)	
H13	0.5616	1.1132	0.1366	0.059*	
C14	0.5390 (5)	1.2602 (4)	0.2373 (4)	0.0504 (11)	
C15	0.4540 (5)	1.3103 (3)	0.2983 (3)	0.0414 (10)	
H15	0.4763	1.3876	0.3404	0.050*	
C16	0.3379 (5)	1.2431 (3)	0.2945 (3)	0.0404 (10)	
C17	0.2583 (5)	1.3048 (3)	0.3594 (3)	0.0432 (11)	
H17	0.2912	1.3828	0.3947	0.052*	
C18	0.0773 (6)	1.3532 (4)	0.4257 (5)	0.0617 (14)	
H18A	0.0496	1.3860	0.3771	0.074*	
H18B	0.1442	1.4123	0.4846	0.074*	
C19	−0.0426 (6)	1.3263 (4)	0.4654 (5)	0.0640 (14)	
H19A	−0.0424	1.3921	0.5265	0.077*	
H19B	−0.1235	1.3116	0.4124	0.077*	
C20	−0.0452 (6)	1.2255 (4)	0.4931 (4)	0.0636 (14)	
H20A	−0.1168	1.2212	0.5273	0.076*	
H20B	0.0381	1.2398	0.5435	0.076*	
C21	−0.1399 (5)	1.0223 (3)	0.4035 (3)	0.0440 (11)	
H21	−0.1872	1.0383	0.4544	0.053*	
C22	0.1495 (4)	0.6856 (3)	0.2501 (3)	0.0371 (9)	
H22A	0.2457	0.7103	0.2782	0.044*	
H22B	0.1092	0.7226	0.3061	0.044*	
C23	0.1124 (4)	0.5656 (3)	0.2207 (3)	0.0390 (9)	
C24	−0.0014 (5)	0.5072 (4)	0.2283 (4)	0.0487 (11)	
H24	−0.0718	0.5371	0.2501	0.058*	
C25	0.0068 (5)	0.3975 (4)	0.1981 (4)	0.0561 (12)	
H25	−0.0560	0.3401	0.1992	0.067*	
C26	0.1152 (6)	0.3844 (4)	0.1672 (4)	0.0525 (12)	
H26	0.1430	0.3171	0.1413	0.063*	
C27	0.4391 (4)	0.8521 (4)	0.1754 (3)	0.0388 (9)	
H27A	0.4943	0.9261	0.2236	0.047*	
H27B	0.4217	0.8090	0.2159	0.047*	
C28	0.5147 (4)	0.7964 (4)	0.1059 (3)	0.0398 (9)	
C29	0.6172 (5)	0.8350 (5)	0.0721 (4)	0.0562 (12)	
H29	0.6626	0.9103	0.0965	0.067*	
C30	0.6425 (5)	0.7448 (5)	−0.0037 (4)	0.0631 (15)	
H30	0.7032	0.7477	−0.0434	0.076*	
C31	0.5636 (6)	0.6517 (4)	−0.0096 (4)	0.0615 (14)	
H31	0.5643	0.5773	−0.0503	0.074*	
C32	0.5514 (6)	0.1983 (4)	0.4781 (4)	0.0675 (16)	
H32A	0.5889	0.1653	0.5229	0.101*	
H32B	0.5626	0.1591	0.4095	0.101*	
H32C	0.4578	0.1922	0.4748	0.101*	
C33	0.6194 (6)	0.3177 (5)	0.5190 (5)	0.0705 (16)	
Cl1	−0.11526 (12)	1.10354 (8)	0.15969 (8)	0.0413 (2)	
Cl2	0.30948 (13)	0.95565 (9)	0.38563 (8)	0.0497 (3)	
Cl3	−0.42396 (18)	0.62659 (12)	0.29107 (15)	0.0837 (5)	
Cl4	0.68796 (15)	1.34258 (11)	0.24439 (13)	0.0706 (4)	
Cu1	0.05232 (6)	1.10190 (4)	0.30743 (4)	0.03763 (14)	
Cu2	0.17812 (5)	0.90346 (4)	0.21979 (4)	0.03578 (14)	
N1	0.1122 (4)	0.7269 (3)	0.1669 (2)	0.0364 (8)	
N2	0.3080 (3)	0.8683 (3)	0.1268 (2)	0.0345 (7)	
N3	0.1460 (4)	1.2632 (3)	0.3737 (3)	0.0502 (10)	
N4	−0.0640 (5)	1.1100 (3)	0.4042 (3)	0.0547 (11)	
N5	0.6739 (6)	0.4137 (4)	0.5554 (4)	0.0875 (17)	
O1	0.0144 (3)	0.9354 (2)	0.2580 (2)	0.0398 (7)	
O2	0.1891 (3)	1.06154 (19)	0.22835 (19)	0.0350 (6)	
O3	0.1847 (3)	0.4960 (3)	0.1811 (2)	0.0505 (8)	
O4	0.4820 (3)	0.6849 (3)	0.0544 (3)	0.0586 (9)	

### Atomic displacement parameters (Å^2^)

**Table:**

	*U*^11^	*U*^22^	*U*^33^	*U*^12^	*U*^13^	*U*^23^
C1	0.051 (2)	0.040 (2)	0.0213 (18)	0.0135 (18)	0.0152 (17)	0.0188 (16)
C2	0.062 (3)	0.036 (2)	0.033 (2)	0.022 (2)	0.029 (2)	0.0204 (17)
C3	0.063 (3)	0.054 (3)	0.061 (3)	0.012 (2)	0.034 (3)	0.032 (2)
C4	0.058 (3)	0.049 (3)	0.068 (3)	0.015 (2)	0.031 (3)	0.036 (2)
C5	0.072 (3)	0.028 (2)	0.050 (3)	0.010 (2)	0.023 (2)	0.0145 (19)
C6	0.058 (3)	0.043 (2)	0.026 (2)	0.015 (2)	0.0180 (19)	0.0175 (17)
C7	0.057 (3)	0.035 (2)	0.0240 (19)	0.0244 (19)	0.0156 (18)	0.0140 (15)
C8	0.066 (3)	0.0089 (15)	0.037 (2)	0.0089 (16)	0.021 (2)	0.0045 (14)
C9	0.060 (3)	0.0257 (18)	0.0210 (17)	0.0159 (17)	0.0191 (17)	0.0096 (14)
C10	0.052 (2)	0.041 (2)	0.0163 (16)	0.0254 (19)	0.0118 (16)	0.0168 (15)
C11	0.048 (2)	0.0252 (18)	0.035 (2)	0.0149 (17)	0.0100 (18)	0.0170 (16)
C12	0.056 (3)	0.0266 (18)	0.030 (2)	0.0124 (18)	0.0135 (18)	0.0139 (16)
C13	0.074 (3)	0.041 (2)	0.052 (3)	0.014 (2)	0.036 (2)	0.031 (2)
C14	0.062 (3)	0.043 (2)	0.059 (3)	0.018 (2)	0.022 (2)	0.030 (2)
C15	0.060 (3)	0.0237 (19)	0.047 (2)	0.0096 (19)	0.020 (2)	0.0182 (17)
C16	0.057 (3)	0.029 (2)	0.044 (2)	0.0118 (19)	0.013 (2)	0.0239 (18)
C17	0.080 (3)	0.0129 (16)	0.037 (2)	0.0040 (19)	0.021 (2)	0.0097 (15)
C18	0.086 (4)	0.026 (2)	0.076 (4)	0.017 (2)	0.035 (3)	0.018 (2)
C19	0.076 (4)	0.054 (3)	0.073 (4)	0.030 (3)	0.030 (3)	0.026 (3)
C20	0.077 (4)	0.043 (3)	0.074 (4)	0.022 (3)	0.039 (3)	0.017 (3)
C21	0.082 (3)	0.032 (2)	0.035 (2)	0.025 (2)	0.040 (2)	0.0166 (17)
C22	0.055 (3)	0.0221 (17)	0.030 (2)	−0.0007 (17)	0.0118 (18)	0.0093 (15)
C23	0.055 (3)	0.031 (2)	0.039 (2)	0.0122 (19)	0.013 (2)	0.0219 (17)
C24	0.061 (3)	0.039 (2)	0.056 (3)	0.015 (2)	0.027 (2)	0.023 (2)
C25	0.069 (3)	0.033 (2)	0.065 (3)	0.003 (2)	0.022 (3)	0.019 (2)
C26	0.080 (4)	0.042 (2)	0.045 (3)	0.025 (2)	0.023 (2)	0.020 (2)
C27	0.064 (3)	0.042 (2)	0.0280 (19)	0.023 (2)	0.0159 (19)	0.0272 (17)
C28	0.049 (3)	0.048 (2)	0.035 (2)	0.021 (2)	0.0125 (19)	0.0257 (19)
C29	0.047 (3)	0.074 (3)	0.057 (3)	0.018 (3)	0.015 (2)	0.035 (3)
C30	0.045 (3)	0.086 (4)	0.049 (3)	0.006 (3)	0.018 (2)	0.017 (3)
C31	0.069 (3)	0.051 (3)	0.072 (4)	0.030 (3)	0.035 (3)	0.021 (3)
C32	0.081 (4)	0.039 (3)	0.060 (3)	−0.014 (2)	−0.022 (3)	0.024 (2)
C33	0.078 (4)	0.067 (4)	0.067 (4)	0.024 (3)	0.002 (3)	0.033 (3)
Cl1	0.0676 (7)	0.0326 (5)	0.0326 (5)	0.0175 (5)	0.0229 (5)	0.0163 (4)
Cl2	0.0790 (8)	0.0370 (5)	0.0312 (5)	0.0088 (5)	0.0142 (5)	0.0134 (4)
Cl3	0.0998 (12)	0.0533 (8)	0.1164 (13)	0.0147 (8)	0.0643 (11)	0.0393 (8)
Cl4	0.0689 (8)	0.0489 (7)	0.1056 (12)	0.0101 (6)	0.0410 (8)	0.0372 (8)
Cu1	0.0614 (3)	0.0294 (2)	0.0267 (3)	0.0145 (2)	0.0220 (2)	0.01025 (19)
Cu2	0.0611 (3)	0.0225 (2)	0.0300 (3)	0.0136 (2)	0.0219 (2)	0.01146 (18)
N1	0.058 (2)	0.0252 (16)	0.0264 (16)	0.0156 (15)	0.0164 (15)	0.0060 (13)
N2	0.0452 (19)	0.0303 (17)	0.0317 (17)	0.0143 (15)	0.0119 (15)	0.0137 (14)
N3	0.085 (3)	0.0265 (17)	0.045 (2)	0.0112 (18)	0.033 (2)	0.0137 (15)
N4	0.084 (3)	0.038 (2)	0.052 (2)	0.019 (2)	0.039 (2)	0.0176 (18)
N5	0.109 (4)	0.050 (3)	0.069 (3)	−0.013 (3)	0.000 (3)	0.009 (2)
O1	0.0621 (19)	0.0240 (13)	0.0334 (15)	0.0056 (13)	0.0229 (14)	0.0085 (11)
O2	0.0628 (18)	0.0175 (12)	0.0296 (14)	0.0101 (12)	0.0228 (13)	0.0096 (10)
O3	0.066 (2)	0.0504 (18)	0.0445 (17)	0.0207 (16)	0.0284 (16)	0.0206 (14)
O4	0.062 (2)	0.0493 (19)	0.059 (2)	0.0233 (16)	0.0244 (17)	0.0084 (16)

### Geometric parameters (Å, °)

**Table:**

C1—O1	1.352 (5)	C20—N4	1.524 (6)
C1—C6	1.394 (6)	C20—H20A	0.9700
C1—C2	1.408 (5)	C20—H20B	0.9700
C2—C3	1.385 (6)	C21—N4	1.293 (6)
C2—C21	1.410 (5)	C21—H21	0.9300
C3—C4	1.384 (7)	C22—C23	1.436 (5)
C3—H3	0.9300	C22—N1	1.498 (5)
C4—C5	1.384 (6)	C22—H22A	0.9700
C4—Cl3	1.736 (5)	C22—H22B	0.9700
C5—C6	1.419 (6)	C23—O3	1.298 (5)
C5—H5	0.9300	C23—C24	1.362 (6)
C6—C7	1.514 (5)	C24—C25	1.355 (6)
C7—N1	1.504 (5)	C24—H24	0.9300
C7—H7A	0.9700	C25—C26	1.298 (7)
C7—H7B	0.9700	C25—H25	0.9300
C8—N1	1.449 (5)	C26—O3	1.457 (6)
C8—C9	1.548 (5)	C26—H26	0.9300
C8—H8A	0.9700	C27—C28	1.431 (6)
C8—H8B	0.9700	C27—N2	1.503 (5)
C9—N2	1.448 (5)	C27—H27A	0.9700
C9—H9A	0.9700	C27—H27B	0.9700
C9—H9B	0.9700	C28—O4	1.322 (5)
C10—N2	1.486 (5)	C28—C29	1.359 (6)
C10—C12	1.507 (5)	C29—C30	1.369 (7)
C10—H10A	0.9700	C29—H29	0.9300
C10—H10B	0.9700	C30—C31	1.334 (7)
C11—O2	1.343 (5)	C30—H30	0.9300
C11—C16	1.385 (5)	C31—O4	1.357 (6)
C11—C12	1.394 (5)	C31—H31	0.9300
C12—C13	1.384 (6)	C32—C33	1.460 (8)
C13—C14	1.370 (6)	C32—H32A	0.9600
C13—H13	0.9300	C32—H32B	0.9600
C14—C15	1.414 (6)	C32—H32C	0.9600
C14—Cl4	1.724 (5)	C33—N5	1.171 (7)
C15—C16	1.371 (6)	Cu1—Cl1	2.5022 (13)
C15—H15	0.9300	Cu1—O1	1.974 (3)
C16—C17	1.453 (6)	Cu1—O2	1.987 (3)
C17—N3	1.304 (6)	Cu1—N3	1.971 (4)
C17—H17	0.9300	Cu1—N4	1.984 (4)
C18—N3	1.494 (6)	Cu2—Cl2	2.3104 (13)
C18—C19	1.513 (7)	Cu2—O1	1.940 (3)
C18—H18A	0.9700	Cu2—O2	2.010 (2)
C18—H18B	0.9700	Cu2—N1	2.104 (3)
C19—C20	1.511 (7)	Cu2—N2	2.047 (3)
C19—H19A	0.9700	Cu1—Cu2	3.0274 (9)
C19—H19B	0.9700		
			
O1—C1—C6	119.1 (3)	C24—C23—C22	126.5 (4)
O1—C1—C2	120.7 (4)	C25—C24—C23	107.5 (4)
C6—C1—C2	120.2 (4)	C25—C24—H24	126.3
C3—C2—C1	120.4 (4)	C23—C24—H24	126.3
C3—C2—C21	116.9 (4)	C26—C25—C24	109.8 (5)
C1—C2—C21	122.7 (4)	C26—C25—H25	125.1
C4—C3—C2	119.5 (4)	C24—C25—H25	125.1
C4—C3—H3	120.3	C25—C26—O3	106.5 (4)
C2—C3—H3	120.3	C25—C26—H26	126.8
C5—C4—C3	121.3 (4)	O3—C26—H26	126.8
C5—C4—Cl3	118.9 (4)	C28—C27—N2	116.7 (3)
C3—C4—Cl3	119.8 (4)	C28—C27—H27A	108.1
C4—C5—C6	119.9 (4)	N2—C27—H27A	108.1
C4—C5—H5	120.1	C28—C27—H27B	108.1
C6—C5—H5	120.1	N2—C27—H27B	108.1
C1—C6—C5	118.7 (4)	H27A—C27—H27B	107.3
C1—C6—C7	122.4 (4)	O4—C28—C29	106.5 (4)
C5—C6—C7	118.7 (4)	O4—C28—C27	120.3 (4)
N1—C7—C6	113.0 (3)	C29—C28—C27	133.1 (4)
N1—C7—H7A	109.0	C28—C29—C30	108.7 (5)
C6—C7—H7A	109.0	C28—C29—H29	125.7
N1—C7—H7B	109.0	C30—C29—H29	125.7
C6—C7—H7B	109.0	C31—C30—C29	106.9 (5)
H7A—C7—H7B	107.8	C31—C30—H30	126.6
N1—C8—C9	111.5 (3)	C29—C30—H30	126.6
N1—C8—H8A	109.3	C30—C31—O4	107.8 (4)
C9—C8—H8A	109.3	C30—C31—H31	126.1
N1—C8—H8B	109.3	O4—C31—H31	126.1
C9—C8—H8B	109.3	C33—C32—H32A	109.5
H8A—C8—H8B	108.0	C33—C32—H32B	109.5
N2—C9—C8	110.5 (3)	H32A—C32—H32B	109.5
N2—C9—H9A	109.5	C33—C32—H32C	109.5
C8—C9—H9A	109.5	H32A—C32—H32C	109.5
N2—C9—H9B	109.5	H32B—C32—H32C	109.5
C8—C9—H9B	109.5	N5—C33—C32	177.6 (7)
H9A—C9—H9B	108.1	N3—Cu1—O1	162.13 (16)
N2—C10—C12	117.2 (3)	N3—Cu1—N4	96.87 (15)
N2—C10—H10A	108.0	O1—Cu1—N4	90.00 (13)
C12—C10—H10A	108.0	N3—Cu1—O2	91.60 (13)
N2—C10—H10B	108.0	O1—Cu1—O2	77.66 (10)
C12—C10—H10B	108.0	N4—Cu1—O2	162.26 (14)
H10A—C10—H10B	107.2	N3—Cu1—Cl1	99.50 (12)
O2—C11—C16	121.8 (4)	O1—Cu1—Cl1	95.76 (9)
O2—C11—C12	119.0 (3)	N4—Cu1—Cl1	98.40 (14)
C16—C11—C12	119.2 (4)	O2—Cu1—Cl1	95.51 (9)
C13—C12—C11	119.9 (4)	N3—Cu1—Cu2	125.95 (12)
C13—C12—C10	120.2 (4)	O1—Cu1—Cu2	38.94 (8)
C11—C12—C10	119.0 (4)	N4—Cu1—Cu2	123.12 (11)
C14—C13—C12	120.7 (4)	O2—Cu1—Cu2	41.06 (7)
C14—C13—H13	119.6	Cl1—Cu1—Cu2	107.70 (3)
C12—C13—H13	119.6	O1—Cu2—O2	77.90 (11)
C13—C14—C15	119.9 (4)	O1—Cu2—N2	157.52 (13)
C13—C14—Cl4	120.6 (4)	O2—Cu2—N2	94.21 (11)
C15—C14—Cl4	119.5 (4)	O1—Cu2—N1	94.20 (12)
C16—C15—C14	118.9 (4)	O2—Cu2—N1	160.26 (13)
C16—C15—H15	120.6	N2—Cu2—N1	86.31 (13)
C14—C15—H15	120.6	O1—Cu2—Cl2	97.67 (9)
C15—C16—C11	121.5 (4)	O2—Cu2—Cl2	96.32 (8)
C15—C16—C17	113.6 (4)	N2—Cu2—Cl2	104.15 (10)
C11—C16—C17	124.9 (4)	N1—Cu2—Cl2	102.70 (10)
N3—C17—C16	127.4 (3)	O1—Cu2—Cu1	39.76 (8)
N3—C17—H17	116.3	O2—Cu2—Cu1	40.49 (7)
C16—C17—H17	116.3	N2—Cu2—Cu1	134.50 (9)
N3—C18—C19	121.2 (4)	N1—Cu2—Cu1	133.94 (10)
N3—C18—H18A	107.0	Cl2—Cu2—Cu1	88.50 (3)
C19—C18—H18A	107.0	C8—N1—C22	110.7 (3)
N3—C18—H18B	107.0	C8—N1—C7	109.0 (3)
C19—C18—H18B	107.0	C22—N1—C7	112.1 (3)
H18A—C18—H18B	106.8	C8—N1—Cu2	105.5 (2)
C20—C19—C18	113.4 (4)	C22—N1—Cu2	113.0 (2)
C20—C19—H19A	108.9	C7—N1—Cu2	106.2 (2)
C18—C19—H19A	108.9	C9—N2—C10	110.4 (3)
C20—C19—H19B	108.9	C9—N2—C27	113.1 (3)
C18—C19—H19B	108.9	C10—N2—C27	107.8 (3)
H19A—C19—H19B	107.7	C9—N2—Cu2	99.3 (2)
C19—C20—N4	117.2 (4)	C10—N2—Cu2	110.3 (2)
C19—C20—H20A	108.0	C27—N2—Cu2	115.6 (2)
N4—C20—H20A	108.0	C17—N3—C18	112.3 (4)
C19—C20—H20B	108.0	C17—N3—Cu1	125.0 (3)
N4—C20—H20B	108.0	C18—N3—Cu1	121.2 (3)
H20A—C20—H20B	107.2	C21—N4—C20	119.3 (4)
N4—C21—C2	130.5 (4)	C21—N4—Cu1	123.8 (3)
N4—C21—H21	114.7	C20—N4—Cu1	116.2 (3)
C2—C21—H21	114.7	C1—O1—Cu2	127.6 (2)
C23—C22—N1	117.1 (3)	C1—O1—Cu1	129.8 (2)
C23—C22—H22A	108.0	Cu2—O1—Cu1	101.30 (12)
N1—C22—H22A	108.0	C11—O2—Cu1	129.2 (2)
C23—C22—H22B	108.0	C11—O2—Cu2	123.0 (2)
N1—C22—H22B	108.0	Cu1—O2—Cu2	98.45 (11)
H22A—C22—H22B	107.3	C23—O3—C26	106.4 (3)
O3—C23—C24	109.7 (4)	C28—O4—C31	109.9 (4)
O3—C23—C22	123.8 (4)		
			
O1—C1—C2—C3	179.0 (4)	C8—C9—N2—C10	−170.1 (3)
C6—C1—C2—C3	0.7 (6)	C8—C9—N2—C27	68.9 (4)
O1—C1—C2—C21	−2.5 (6)	C8—C9—N2—Cu2	−54.2 (3)
C6—C1—C2—C21	179.3 (4)	C12—C10—N2—C9	162.6 (3)
C1—C2—C3—C4	−1.5 (7)	C12—C10—N2—C27	−73.3 (4)
C21—C2—C3—C4	179.9 (5)	C12—C10—N2—Cu2	53.8 (4)
C2—C3—C4—C5	1.4 (8)	C28—C27—N2—C9	48.9 (5)
C2—C3—C4—Cl3	−177.7 (4)	C28—C27—N2—C10	−73.5 (4)
C3—C4—C5—C6	−0.6 (8)	C28—C27—N2—Cu2	162.5 (3)
Cl3—C4—C5—C6	178.5 (4)	O1—Cu2—N2—C9	−57.1 (4)
O1—C1—C6—C5	−178.2 (4)	O2—Cu2—N2—C9	−125.3 (2)
C2—C1—C6—C5	0.0 (6)	N1—Cu2—N2—C9	34.9 (2)
O1—C1—C6—C7	−3.1 (6)	Cl2—Cu2—N2—C9	137.1 (2)
C2—C1—C6—C7	175.2 (4)	Cu1—Cu2—N2—C9	−120.8 (2)
C4—C5—C6—C1	−0.1 (7)	O1—Cu2—N2—C10	58.9 (4)
C4—C5—C6—C7	−175.4 (4)	O2—Cu2—N2—C10	−9.3 (3)
C1—C6—C7—N1	58.5 (5)	N1—Cu2—N2—C10	150.9 (3)
C5—C6—C7—N1	−126.4 (4)	Cl2—Cu2—N2—C10	−106.9 (2)
N1—C8—C9—N2	51.4 (5)	Cu1—Cu2—N2—C10	−4.8 (3)
O2—C11—C12—C13	−179.0 (4)	O1—Cu2—N2—C27	−178.5 (3)
C16—C11—C12—C13	−0.5 (6)	O2—Cu2—N2—C27	113.4 (3)
O2—C11—C12—C10	12.2 (5)	N1—Cu2—N2—C27	−86.4 (3)
C16—C11—C12—C10	−169.3 (3)	Cl2—Cu2—N2—C27	15.8 (3)
N2—C10—C12—C13	126.0 (4)	Cu1—Cu2—N2—C27	117.9 (2)
N2—C10—C12—C11	−65.1 (5)	C16—C17—N3—C18	−167.2 (4)
C11—C12—C13—C14	−0.3 (7)	C16—C17—N3—Cu1	−1.0 (7)
C10—C12—C13—C14	168.4 (4)	C19—C18—N3—C17	−170.1 (5)
C12—C13—C14—C15	0.7 (7)	C19—C18—N3—Cu1	23.1 (7)
C12—C13—C14—Cl4	178.4 (4)	O1—Cu1—N3—C17	50.6 (7)
C13—C14—C15—C16	−0.2 (7)	N4—Cu1—N3—C17	162.6 (4)
Cl4—C14—C15—C16	−177.9 (3)	O2—Cu1—N3—C17	−1.8 (4)
C14—C15—C16—C11	−0.6 (6)	Cl1—Cu1—N3—C17	−97.7 (4)
C14—C15—C16—C17	−179.1 (4)	Cu2—Cu1—N3—C17	22.5 (5)
O2—C11—C16—C15	179.4 (4)	O1—Cu1—N3—C18	−144.3 (4)
C12—C11—C16—C15	0.9 (6)	N4—Cu1—N3—C18	−32.4 (4)
O2—C11—C16—C17	−2.2 (6)	O2—Cu1—N3—C18	163.2 (4)
C12—C11—C16—C17	179.3 (4)	Cl1—Cu1—N3—C18	67.4 (4)
C15—C16—C17—N3	−177.8 (4)	Cu2—Cu1—N3—C18	−172.5 (3)
C11—C16—C17—N3	3.7 (7)	C2—C21—N4—C20	−172.4 (5)
N3—C18—C19—C20	26.6 (8)	C2—C21—N4—Cu1	−1.7 (8)
C18—C19—C20—N4	−66.3 (7)	C19—C20—N4—C21	−140.0 (5)
C3—C2—C21—N4	−170.7 (5)	C19—C20—N4—Cu1	48.6 (6)
C1—C2—C21—N4	10.6 (8)	N3—Cu1—N4—C21	−172.5 (4)
N1—C22—C23—O3	86.0 (5)	O1—Cu1—N4—C21	−9.0 (4)
N1—C22—C23—C24	−93.4 (5)	O2—Cu1—N4—C21	−54.5 (8)
O3—C23—C24—C25	4.2 (6)	Cl1—Cu1—N4—C21	86.8 (4)
C22—C23—C24—C25	−176.4 (4)	Cu2—Cu1—N4—C21	−30.8 (5)
C23—C24—C25—C26	−3.4 (6)	N3—Cu1—N4—C20	−1.5 (4)
C24—C25—C26—O3	1.5 (6)	O1—Cu1—N4—C20	161.9 (4)
N2—C27—C28—O4	−78.7 (5)	O2—Cu1—N4—C20	116.5 (5)
N2—C27—C28—C29	96.3 (6)	Cl1—Cu1—N4—C20	−102.2 (4)
O4—C28—C29—C30	2.2 (5)	Cu2—Cu1—N4—C20	140.2 (3)
C27—C28—C29—C30	−173.3 (5)	C6—C1—O1—Cu2	−31.0 (5)
C28—C29—C30—C31	−4.9 (6)	C2—C1—O1—Cu2	150.7 (3)
C29—C30—C31—O4	5.7 (6)	C6—C1—O1—Cu1	164.7 (3)
N3—Cu1—Cu2—O1	166.69 (19)	C2—C1—O1—Cu1	−13.6 (5)
N4—Cu1—Cu2—O1	36.2 (2)	O2—Cu2—O1—C1	175.7 (3)
O2—Cu1—Cu2—O1	−154.57 (18)	N2—Cu2—O1—C1	104.5 (4)
Cl1—Cu1—Cu2—O1	−76.86 (13)	N1—Cu2—O1—C1	14.0 (3)
N3—Cu1—Cu2—O2	−38.75 (18)	Cl2—Cu2—O1—C1	−89.5 (3)
O1—Cu1—Cu2—O2	154.57 (18)	Cu1—Cu2—O1—C1	−167.8 (4)
N4—Cu1—Cu2—O2	−169.3 (2)	O2—Cu2—O1—Cu1	−16.57 (11)
Cl1—Cu1—Cu2—O2	77.71 (13)	N2—Cu2—O1—Cu1	−87.8 (3)
N3—Cu1—Cu2—N2	−45.7 (2)	N1—Cu2—O1—Cu1	−178.28 (13)
O1—Cu1—Cu2—N2	147.61 (19)	Cl2—Cu2—O1—Cu1	78.29 (11)
N4—Cu1—Cu2—N2	−176.2 (2)	N3—Cu1—O1—C1	129.9 (5)
O2—Cu1—Cu2—N2	−6.96 (18)	N4—Cu1—O1—C1	17.0 (3)
Cl1—Cu1—Cu2—N2	70.75 (14)	O2—Cu1—O1—C1	−175.9 (3)
N3—Cu1—Cu2—N1	169.07 (18)	Cl1—Cu1—O1—C1	−81.5 (3)
O1—Cu1—Cu2—N1	2.38 (18)	Cu2—Cu1—O1—C1	167.4 (4)
N4—Cu1—Cu2—N1	38.6 (2)	N3—Cu1—O1—Cu2	−37.4 (5)
O2—Cu1—Cu2—N1	−152.19 (17)	N4—Cu1—O1—Cu2	−150.37 (17)
Cl1—Cu1—Cu2—N1	−74.48 (13)	O2—Cu1—O1—Cu2	16.78 (12)
N3—Cu1—Cu2—Cl2	62.80 (14)	Cl1—Cu1—O1—Cu2	111.19 (10)
O1—Cu1—Cu2—Cl2	−103.89 (13)	C16—C11—O2—Cu1	−1.7 (5)
N4—Cu1—Cu2—Cl2	−67.72 (16)	C12—C11—O2—Cu1	176.8 (3)
O2—Cu1—Cu2—Cl2	101.54 (13)	C16—C11—O2—Cu2	−140.9 (3)
Cl1—Cu1—Cu2—Cl2	179.25 (4)	C12—C11—O2—Cu2	37.6 (5)
C9—C8—N1—C22	−139.9 (3)	N3—Cu1—O2—C11	3.2 (3)
C9—C8—N1—C7	96.4 (4)	O1—Cu1—O2—C11	−162.4 (3)
C9—C8—N1—Cu2	−17.3 (4)	N4—Cu1—O2—C11	−115.5 (5)
C23—C22—N1—C8	−60.1 (5)	Cl1—Cu1—O2—C11	102.9 (3)
C23—C22—N1—C7	61.8 (5)	Cu2—Cu1—O2—C11	−146.3 (4)
C23—C22—N1—Cu2	−178.1 (3)	N3—Cu1—O2—Cu2	149.55 (15)
C6—C7—N1—C8	−179.5 (3)	O1—Cu1—O2—Cu2	−16.04 (12)
C6—C7—N1—C22	57.6 (4)	N4—Cu1—O2—Cu2	30.8 (5)
C6—C7—N1—Cu2	−66.3 (3)	Cl1—Cu1—O2—Cu2	−110.75 (9)
O1—Cu2—N1—C8	147.6 (3)	O1—Cu2—O2—C11	165.5 (3)
O2—Cu2—N1—C8	82.3 (4)	N2—Cu2—O2—C11	−35.8 (3)
N2—Cu2—N1—C8	−9.9 (3)	N1—Cu2—O2—C11	−126.5 (4)
Cl2—Cu2—N1—C8	−113.6 (3)	Cl2—Cu2—O2—C11	69.0 (3)
Cu1—Cu2—N1—C8	146.1 (2)	Cu1—Cu2—O2—C11	149.2 (3)
O1—Cu2—N1—C22	−91.3 (3)	O1—Cu2—O2—Cu1	16.31 (12)
O2—Cu2—N1—C22	−156.6 (3)	N2—Cu2—O2—Cu1	175.03 (13)
N2—Cu2—N1—C22	111.2 (3)	N1—Cu2—O2—Cu1	84.2 (3)
Cl2—Cu2—N1—C22	7.5 (3)	Cl2—Cu2—O2—Cu1	−80.21 (10)
Cu1—Cu2—N1—C22	−92.9 (3)	C24—C23—O3—C26	−3.2 (5)
O1—Cu2—N1—C7	32.0 (2)	C22—C23—O3—C26	177.4 (4)
O2—Cu2—N1—C7	−33.3 (5)	C25—C26—O3—C23	1.1 (5)
N2—Cu2—N1—C7	−125.4 (2)	C29—C28—O4—C31	1.4 (5)
Cl2—Cu2—N1—C7	130.9 (2)	C27—C28—O4—C31	177.6 (4)
Cu1—Cu2—N1—C7	30.5 (3)	C30—C31—O4—C28	−4.5 (6)

## References

[bb1] Bruker (2001). *SADABS* Bruker AXS Inc., Madison, Wisconsin, USA.

[bb2] Bruker (2007). *SMART* and *SAINT* Bruker AXS Inc., Madison, Wisconsin, USA.

[bb3] Hori, A., Yonemura, M., Ohba, M. & Okawa, H. (2001). *Bull. Chem. Soc. Jpn*, **74**, 495–503.

[bb4] Karunakaran, S. & Kandaswamy, M. (1994). *J. Chem. Soc. Dalton Trans.* pp. 1595–1598.

[bb5] McCollum, D. G., Fraser, C., Ostrander, R., Rheingold, A. L. & Bosnich, B. (1994). *Inorg. Chem.* **33**, 2383–2392.

[bb6] Okawa, H., Furutachi, H. & Fenton, D. E. (1998). *Coord. Chem. Rev.* **174**, 51–75.

[bb7] Rameau, J. Th. L. B. (1938). *Rev. Trav. Chim.* **57**, 192–214.

[bb8] Sheldrick, G. M. (2008). *Acta Cryst.* A**64**, 112–122. 10.1107/S0108767307043930 18156677

[bb9] Sun, G.-C., He, Z.-H., Li, Z.-J., Yuan, X.-D., Yang, Z.-J., Wang, G.-X., Wang, L.-F. & Liu, C.-R. (2001). *Molecules*, **6**, 1001–1005.

